# Current Use and Effects of Osteopathic Manipulative Treatment (OMT) in the Military: A Scoping Review

**DOI:** 10.7759/cureus.79844

**Published:** 2025-02-28

**Authors:** Heather Lumley, Nozimakhon Omonullaeva, Philip Dainty, Joseph Paquette, Jack Stensland, Kelsey Reindel

**Affiliations:** 1 Osteopathic Medicine, Nova Southeastern University Dr. Kiran C. Patel College of Osteopathic Medicine, Clearwater, USA; 2 Osteopathic Medicine, Nova Southeastern University Dr. Kiran C. Patel College of Osteopathic Medicine, Davie, USA

**Keywords:** military, musculoskeletal, osteopathic manipulative medicine, osteopathic manipulative treatment, pain management, veterans

## Abstract

Osteopathic manipulative treatment (OMT) is a hands-on therapy approach used by osteopathic physicians that aims to alleviate viscero-somatic changes by considering the interconnectedness of the body, mind, and spirit. One of the most common uses for OMT is the treatment of musculoskeletal (MSK) complaints, which are a significant cause of pain and disability across various populations, including the US Military, where personnel often face unique and physically demanding working conditions. In 2019, MSK injuries alone accounted for nearly half of the limited duty days in the US Army, with low back pain being the leading cause of medical encounters over the past 10 years among active-duty personnel. Previous studies have shown that OMT can improve functional status, reduce pain, and have minimal adverse effects when treating low back pain. It has also been found to be twice as effective as placebo treatments and comparable to nonsteroidal anti-inflammatory drugs in terms of pain relief. Given the high incidence rates of MSK issues during active duty in the military, OMT may be a viable treatment or adjunctive therapy option for this population. The purpose of this scoping review is to evaluate the current research on OMT among US military members and veterans, determining any potential positive or negative effects that may be attributed to this therapy. A comprehensive search was conducted across six databases from inception to November 2023, yielding 497 articles, which were screened according to inclusion and exclusion criteria. Nine studies were ultimately included and evaluated based on the target treatment area. The most commonly employed forms of OMT included myofascial release, soft tissue manipulation, and counterstrain techniques. The primary outcomes after OMT were reduced pain and improved range of motion or functionality. Other positive findings included a decrease in opioid usage, reduced nausea, lower healthcare costs, and lower incidence of alcohol and substance use disorders within the military populations studied. This is the first review of its kind to evaluate OMT within military populations, demonstrating its effectiveness in reducing pain, increasing functionality, and lowering complications associated with military duties. Further research into the utilization of OMT within the military should be pursued, considering it as a potential first-line or adjunctive therapy for service members and veterans across various settings and dysfunctions to continue evaluating its long-term effectiveness.

## Introduction and background

Osteopathic manipulative treatment (OMT) is a set of treatment modalities developed by the Doctors of Osteopathic Medicine (DOs) and has been established since its inception in 1874 by A.T. Still University [1]. OMT is a component of the broader field of osteopathic manipulative medicine (OMM), which aims to address and correct dysfunctions in various body systems, including musculoskeletal (MSK), cranial, nervous, hematological, and lymphatic systems. OMM emphasizes a holistic approach to patient care, striving to maintain equilibrium in the body, mind, and spirit [2]. In many cases, dysfunctions in the body correlate with impaired functions and may manifest as observable changes, termed viscero-somatic dysfunctions [3]. These dysfunctions are assessed through the TART changes, an osteopathic acronym meaning tissue texture changes, asymmetry, restriction of motion, and tenderness [3]. Such changes can lead to secondary effects in other interconnected areas of the body. When performing OMT, patients are initially evaluated by assessing range of motion (ROM), pain levels, hypertonicity, and either physiological or anatomical restriction. Treatment modalities used by the DOs are varied and can be categorized as either active or passive (depending on the patient's involvement) and direct or indirect (based on whether treatment is directed toward or away from the palpated restriction).

An increasing body of research highlights the positive effects of OMT on patient care. A meta-analysis in 2014 found that OMT improved functional status, reduced pain, and had minimal adverse effects in the treatment of low back pain [4]. Other studies have shown that OMT shows promise in postoperative patients by reducing pain and minimizing opioid use. It has also been shown to be twice as effective as placebo treatment, delivering analgesic effects comparable to those of nonsteroidal anti-inflammatory drugs [5,6]. As an adjuvant to usual care, OMT has been found to reduce back pain for 3-12 months following treatment, lowering the use of over-the-counter drugs during that period [7]. OMT also helps correct gait asymmetries, particularly by targeting the innominate, sacrum, and lumbar spine, which helps prevent MSK overuse injuries [8]. Additionally, patients who receive standard care for low back pain supplemented by OMT are less likely to require opioid prescriptions, interventional therapy, or radiology services [9].

One of the most common uses of OMT is for MSK complaints, which are a significant cause of pain and disability in various populations, including the US Military, where personnel often face unique and physically demanding working conditions. In 2019, MSK injuries alone accounted for 65.3% of limited duty days for men and 41.9% for women in the US Army, with low back pain being the most common medical encounter over the past 10 years among active-duty personnel [10,11]. As of 2023, noncombative MSK injuries have accounted for 76% of the US military’s active-duty, non-deployable force injuries, making MSK injuries the leading cause of healthcare utilization and disability in the US military [12]. According to a 2020 study, MSK injuries are responsible for 60% of limited duty days and 65% of cases where soldiers cannot deploy for medical reasons within the US active-duty military population [13].

This article was previously presented as a poster at the Association of Military Osteopathic Physicians and Surgeons Annual Conference in Tulsa, OK, on March 2, 2024, and at the Florida Academy of Osteopathy Scientific Research Competition on May 16, 2024.

## Review

Methods

Identifying the Research Question

Our research question was developed based on the Population, Concept and Context (PCC) strategy: population included patients of any age, concept included patients with active military service or veteran status in the setting of a hospital, clinic, or rehabilitation facility, and context included the usage of OMT on patients. Using this strategy, the review question was identified as follows: "What is the current literature showing about the usage and effects of OMT in military members?"

Identifying Relevant Studies

A search was conducted across several databases, including Embase, Ovid MEDLINE, Web of Science, CINAHL, Cochrane Central Register of Controlled Trials, and SPORTDiscus, with full-text access, to include citations from inception to November 28, 2023. Authors HL and JP each performed an initial search independently, using the same controlled terms, as outlined in Table 1, to broaden the search and ensure consistency. To account for variations in terminology, the Boolean operators "AND" (for the simultaneous occurrence of subjects) and "OR" (for the occurrence of their respective ) synonyms were employed. As shown in Table 1, the Boolean operators were applied as follows: #1 OR #2 OR #3 OR #4 OR #7 OR #8, then #5 OR #6, and then these were subsequently combined with AND.

**Table 1:** Boolean operators used for the search

Search number	Search query
#8	"lymph* drain*":ab,ti,kw OR "lymph* massage*":ab,ti,kw OR "lymph* manual drain*":ab,ti,kw OR myofascial:ab,ti,kw OR "myo fascial":ab,ti,kw OR "massage therap*":ab,ti,kw OR "movement therap*":ab,ti,kw
#7	"manual lymphatic drainage"/exp OR "myofascial release"/exp
#6	military:ab,ti,kw OR army:ab,ti,kw OR veteran*:ab,ti,kw OR "air force":ab,ti,kw OR navy:ab,ti,kw OR naval:ab,ti,kw OR "service member*":ab,ti,kw
#5	"military personnel"/exp OR "military phenomena"/exp OR "veteran"/exp OR "military health"/exp
#4	"musculoskeletal manipulation*":ab,ti,kw OR "musculo skeletal manipulation*":ab,ti,kw OR "soft tissue therap*":ab,ti,kw OR "spine manipulation*":ab,ti,kw OR "orthop*dic manipulation*":ab,ti,kw
#3	"musculoskeletal manipulation"/exp OR "soft tissue therapy"/exp
#2	Osteopath*:ab,ti,kw OR omm:ab,ti,kw OR omt:ab,ti,kw
#1	"osteopathic medicine"/exp

Selecting Studies

Our inclusion criteria specified that all articles must be peer-reviewed, written, and published in English, involve active military members or veterans from any branch of the US Military, and include the use of any OMT techniques. Research studies from any time period were eligible for inclusion. Articles were excluded if they focused solely on chiropractic treatment, physical therapy, or acupuncture or were not conducted by a licensed osteopathic physician or an allopathic physician trained in osteopathic manipulations. Studies involving dependents, inactive military members, or military members outside the United States were also excluded. Additionally, editorial papers, systematic reviews, meta-analysis, or other scoping reviews were excluded. 

Charting the Data

A system to chart relevant variables from the data was created by author HL and used by all authors to assess body region, treatment length, OMT techniques used, number of participants, provider of the treatments, follow-up, and limitations of each included study. The six-member research team divided the articles to extract data, with at least two reviewers examining each article to ensure consistency. A third reviewer was consulted for any articles with discrepancies between the first two reviewers. 

Collating, Summarizing, and Reporting the Results

Quality analysis was conducted by authors HL, PD, and NO using the Joanna Briggs Institute (JBI) Appraisal Tools [14-18]. These assessments were used to evaluate the risk of bias in each article. The JBI Tools categorized the articles into groups based on bias risk: high risk (scoring less than 50%), moderate risk (scoring 50% to 70%), and low risk (scoring more than 70%). Due to the limited number of articles meeting the inclusion criteria, the decision was made to include articles with low and moderate risk of bias while excluding articles with high risk. 

Studies were grouped based on the target treatment area, including the cervical spine, thoracic/lumbar spine, full body, and generalized use of OMT. The information from the articles was summarized narratively to describe the OMT treatments, study population, usage, and outcomes. 

Results

Selection of Sources of Evidence

**Figure 1:**
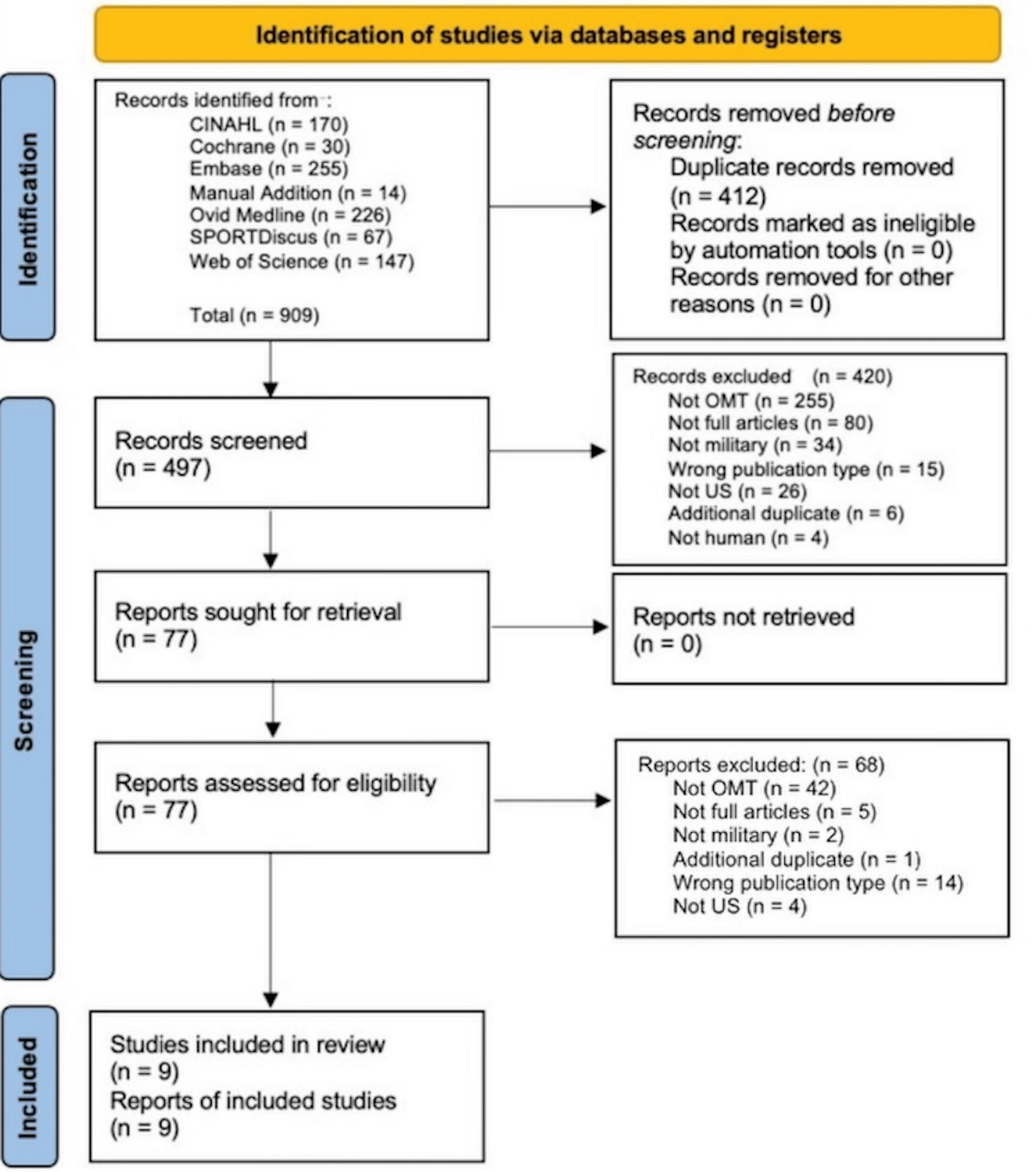
PRISMA-ScR flow diagram

The initial search identified 909 articles related to OMT, MSK manipulation, lymphatic drainage, and military populations, with no limitations applied. After deduplication, 412 articles were removed, leaving 497 articles for screening at the title/abstract level by all six independent blind reviewers. From this screening, 420 articles were excluded based on the following criteria: 255 did not utilize OMT, 80 were not full peer-reviewed articles, 34 did not involve active military members or veterans, 15 were the wrong publication type, 26 were not from the United States, 4 were non-human studies, and 6 were duplicates. Subsequently, 77 full-text articles were screened by two reviewers, with a third reviewer involved in cases of dispute regarding inclusion. Of these, 68 were excluded for the following reasons: 42 did not utilize OMT, 5 were not peer-reviewed full-text articles, 2 did not involve military populations, 14 were the wrong publication type, 4 were not from the United States, and 1 was a duplicate. After screening and exclusions, nine studies remained that met all criteria and were included. The Preferred Reporting Items for Systematic Review and Meta Analysis Protocols extension for Scoping Reviews (PRISMA-ScR) 2020 form was used as a protocol to narrow down and report the nine articles evaluating the use of OMT in military populations, as shown in Figure 1 [19].

Critical Appraisal Within Sources of Evidence

Each article was analyzed using the JBI Appraisal Tool to assess the study type and relevant risk of bias [14-18]. Articles within the moderate risk or low risk of bias were accepted, provided met the inclusion criteria outlined for the study. The appraisal percentages and risk of bias for each included article are shown in Table 2. 

**Table 2:** JBI critical appraisal and risk of bias

Author	Study type	Appraisal %	Risk of bias
Sinkiewicz and Huang [20]	Case series (15)	90%	Low
Thomas et al. [21]	RCT (18)	69%	Moderate
Cruser et al. [22]	RCT (18)	77%	Low
Lipton and McLeod [23]	Case report (14)	88%	Low
Andicochea et al. [24]	Case report (14)	75%	Low
Rhon et al. [25]	Cohort study (17)	50%	Moderate
Rhon et al. [26]	Cohort study (17)	83%	Low
Williams et al. [27]	Cohort study (17)	83%	Low
Meerwijk et al. [28]	Cohort study (17)	92%	Low

A total of nine studies were included in the final review, which were divided into subcategories based on their treatment locations: cervical spine, thoracic/lumbar spine, shoulder and spine, and generalized/full body. A breakdown of the included studies, treatment area, outcomes, and limitations is provided in Table 3. 

**Table 3:** Summary of evidence tx: treatment, sx: symptoms, pt: patient, F: female, M: male, MFR: myofascial release, ME: muscle energy, RCT: randomized controlled trial, CS: counterstrain, HVLA: high-velocity low-amplitude, PT: physical therapist, ER: emergency room, XR: X-ray, CT: computed tomography, MRI: magnetic resonance imaging, CAM: complementary and alternative medicine, NPT: non-pharmacological treatment.

OMT Tx Area	Author and year	Study type	Number of patients and demographics	Type of OMT used	Length of Tx	Outcome and findings	Limitations
Cervical spine	Sinkiewicz and Huang [20]	Case series	n = 8 (3 F, 5 M); ages 25-62; median age 40.5	MFR, soft tissue, ME	At least two OMT tx over a two-week period	Statistically and clinically significant improvements in anxiety and cervicogenic headache pain among patients with mild traumatic brain injury. Clinically significant improvements in 23 of 24 measures of active and passive cervical range of motion	(1) Population size. (2) Results limited by a few numbers of tx sessions
Cervical spine	Thomas et al. [21]	RCT	n = 12 (7 F, 5 M); mean age 29.33	MFR and ME	One tx immediately before experimentally induced motion sickness protocol	Target groups showed lower levels of GI sx (nausea), central sx (dizzy), and sopite-related sx (drowsy), but no significant change was seen in peripheral sx (sweaty)	(1) Population size. (2) Limited blinding efficacy with sham tx. (3) Confounding variables may have impacted the results
Thoracic/lumbar spine	Cruser et al. [22]	RCT	n = 60 OMT tx n = 30 (14 F, 16 M) with a mean age of 26.3 Usual care only tx n = 30 (13 F, 17 M) with a mean age of 27.1	MFR, CS, soft tissue, ME, HVLA, sacro-iliac articulation	One tx per week for four weeks	Statistically significant reduction in pain rating between beginning and end of tx, greater reduction in pain compared to "typical pain," more likely to achieve a 30% pain reduction at an earlier visit, greater reported reduction in "pain at best," and greater satisfaction with tx overall in OMT group compared to usual care only	(1) Confounding variables may have impacted the results. (2) Roland Morris Disability Questionnaire may not accurately assess back pain in active-duty military. (3) No use of a sham group
Thoracic/lumbar spine	Lipton and McLeod [23]	Case report	n = 1 (1 F), age 26	MFR, soft tissue and craniosacral	Around two months	Complete reduction in low back pain with each OMT tx. Once it was determined that the pt had a disc herniation, the pt was referred to neurosurgery, and OMT tx modalities were adjusted to lumbar fascial release and sacral distraction only, but the pt continued to have full pain reduction until surgery	(1) Population size. (2) Unclear number of osteopathic tx sessions
Thoracic/lumbar spine	Andicochea et al. [24]	Case report	n = 1 (1 M), age 45	ME, CS, stretching, and Effleurage	Three days	Pain reduction of 10% and 20% for tx 1 and 2, respectively. Pt reported the day after tx that pain was reduced (80%) and he had no pain when he arrived for tx 3.	(1) Population size
Shoulder and spine	Rhon et al. 25]	Cohort study	n = 7,566	Unspecified	Unspecified	Of the 7,566 pts at the clinic, 2,104 got manual therapy and 1,883 received it in a military clinic. 1,277 were on active duty. The percentage of treatment areas by DOs versus PT and chiropractic were as follows: 30.7% of shoulder diagnoses, 50.6% of cervical diagnoses, 72% of thoracic diagnoses, and 46.4% of lumbar diagnoses that were seen and treated by a physician	(1) Physicians, chiropractors, and PTs performed manual therapy. (2) Confounding variables may not have been accounted for in statistical models. (3) Causality not established due to observational cohort design
Shoulder and spine	Rhon et al. [26]	Cohort study	n = 1,876	Unspecified	Unspecified	From 1,876 unique patients with spine or shoulder disorders receiving manual therapy, 1,162 (61.9%) also received prescription opioids. For pts getting manual therapy first before opioids compared to opioids first, there was a statistically significant lower one-year tx cost, lower mean days’ supply of opioids, and lower mean number of unique opioid prescriptions. Pts with only manual therapy or manual before opioids had significantly fewer visits to the ER and less imaging (XR, CT, or MRI), and had fewer healthcare visits and lower overall healthcare costs. The median time to first opioid for opioid-first was 7 days compared to 158 days in manual therapy-first.	(1) Physicians, chiropractors, and PTs performed manual therapy. (2) Confounding variables may not have been accounted for in statistical models. (3) Causality not established due to observational cohort design
Generalized/full body	Williams et al. [27]	Cohort study	n = 358,394	Unspecified	Unspecified	88% of visits that used a CAM procedure code utilized OMT/chiropractics, and the utilization of OMT/chiropractics more than doubled from 2010 to 2015. Overall, in this population, the use of OMT/chiropractics tended to increase with age and formal education and was more common in women and senior enlisted members or officers. The most frequently labeled diagnosis treated with OMT/chiropractics was “other and unspecified disorders of the back”	(1) Physicians, chiropractors, and PTs performed manual therapy. (2) An unclear number of osteopathic treatment sessions. (3) Unclear types of osteopathic treatments used. (4) Confounding variables due to the use of non-osteopathic techniques
Generalized/full body	Meerwijk et al. [28]	Cohort study	n = 275,820	Unspecified	Unspecified	Active-duty who got NPT for chronic pain were at lower risk for new-onset alcohol or drug use disorder, suicidal ideation, self-inflicted injuries or poisonings (with opioids, narcotics, barbs, or sedatives)	(1) Physicians, chiropractors, and PTs performed manual therapy. (2) Confounding variables due to the use of non-osteopathic techniques. (3) Unclear if the NPT used was for the diagnosed chronic pain. (4) Unclear on specific OMT techniques used. (5) An unclear number of osteopathic treatment sessions

Cervical Spine

For the cervical spine region, the most commonly used OMT techniques were myofascial release (MFR) and muscle energy (ME) [20,21]. In the treatment of the cervical spine, OMT used for cervicogenic headache in patients with mild traumatic brain injury showed clinically significant improvements in 23 of 24 measures of active and passive cervical ROM as well as a significant reduction in pain and anxiety [20]. Cervical OMT was also used to reduce motion sickness in pilots, proving effective for GI and sopite-related symptoms [21]. Sopite-related symptoms, as described and included in the Motion Sickness Assessment Questionnaire (MSAQ), refers to the drowsiness or fatigue that the body experiences due to motion sickness, in addition to the effects on the GI, the central nervous, and the peripheral nervous systems [21].

Thoracic/Lumbar Spine

One randomized controlled trial of OMT for acute low back pain in active-duty personnel found significant reductions in pain, increased satisfaction with treatment, and overall improvement compared to the usual standard of care, such as pharmacological management and physical therapy [22]. The study revealed that subjects receiving OMT were more likely to achieve a statistically significant improvement in "pain at best" at an earlier visit than the usual care-only group [22]. 

In a case report, OMT was used for chronic low back pain in an F-5 pilot. After three treatments using ME, counterstrain, and a combination of stretching and effleurage, the pain diminished to zero on the pain scale. The F-5 pilot was able to maintain his flight status at the conclusion of the study [24].

Another case report involved the use of lumbar high-velocity low-amplitude (HVLA) manipulation to treat a veteran with an initial presentation of low back pain following a recent foot injury. The pain resolved fully after the manipulation. Later, it was discovered that she had a herniated disc; thus, OMT treatment was modified to gentler techniques such as lumbar fascial release and sacral distraction, which continued to alleviate her lower back pain until surgery [23].

Shoulder and Spine

A retrospective observational cohort study evaluated the use of OMT, chiropractic care, and physical therapy manipulations in patients with spine or shoulder dysfunctions. The results showed that 54.5% of the manipulations were performed by chiropractors, 14.4% by both physical therapists and chiropractors, 7.7% by both chiropractors and osteopathic physicians, and 19.0% by other providers [25]. Manipulative treatment utilization varied by diagnosis, with the highest rate (50.2%) in thoracic spine dysfunctions and the lowest rate (20.7%) in shoulder dysfunctions. 

A stepped-care approach to spine and shoulder pain was evaluated, focusing on the use of manual therapy and/or opioids and its impact on healthcare utilization [26]. Of the 1,876 patients with spine and shoulder disorders receiving manual therapy, 61.9% (1,162) were prescribed opioids (453 had OMT first, and 671 had opioids first) [26]. Patients who received OMT first had lower healthcare utilization and costs compared to those in the OMT and opioids group, which saw an average increase of 25.07 outpatient visits and $5,087 higher costs [26]. Overall, the data showed that the group receiving early OMT had the lowest healthcare utilization, while the opioid-use group had the highest [26].

Generalized OMT Usage/Full Body

In a longitudinal study, a six-year surveillance of individuals who served in the active component of the US Military revealed that 14.9% had at least one healthcare encounter involving a complementary and alternative medicine (CAM) procedure, including chiropractic/osteopathic manipulation [27]. CAM procedures are defined as healthcare approaches, practices, and products with a unique history of use outside the realm of Western medicine [27]. Chiropractic/osteopathic manipulation accounted for 88.0% of all CAM encounters. The rates of chiropractic/osteopathic manipulation procedures increased from 2010 to 2015. The most frequent primary diagnoses during CAM-related visits were "other and unspecified disorders of the back" and "nonallopathic lesions (of the MSK system) not elsewhere classified." 

In another longitudinal cohort study, a comparison was made between active-duty US Army service members with chronic pain who received nonpharmacological treatment (NPT) and those who did not receive NPT in the military healthcare system. NPT is an umbrella term for treatment modalities that include exercise therapy, chiropractic, osteopathic spinal manipulation, and less common treatments like yoga, acupuncture, and massage [28]. In this study, OMT was utilized within the context of NPT [28]. The results indicated that active-duty personnel who received NPT were at lower risk of developing alcohol or drug use disorders, suicidal ideation, and self-inflicted injuries/poisonings. Mortality analysis showed a slightly higher mortality rate before any adverse outcomes in the no-NPT group (0.5%) compared to the NPT group (0.4%), with this difference being statistically significant (p < 0.001) [28].

Discussion

Cervical Spine

In the final selection of papers analyzed, the cervical spine was assessed twice, showing statistically significant improvements in multiple modalities, including passive ROM and anxiety reduction [20]. Moreover, their treatment modalities produced significant improvements in 23 out of 24 ROM measures, including both active and passive. Pilots who received OMT for motion sickness reported lower GI and sopite-related symptoms compared to the sham group [21].

Thoracic and Lumbar Spine 

Active-duty personnel reported increased satisfaction and a significant decrease in pain compared to the traditional combination of physical therapy and pharmacologic treatment [22]. A pilot in a case report experienced an 80% reduction in pain after just one treatment, with complete pain resolution that allowed him to remain on active flight status. This was achieved in only three days using ME, counterstrain, and a combination of stretching and effleurage [24]. In another case report, low back pain was resolved after HVLA manipulation. Subsequently, OMT was modified due to surgical indications, yet it continued to provide pain relief up to the date of surgery [23].

Spine and Shoulder

The thoracic spine had the highest percentage of manipulation technique use, while the lumbar spine was the most commonly treated sole region [25]. Patients who received OMT only had lower healthcare utilization and costs, in addition to using outpatient services and opioid prescriptions less frequently [26].

Generalized OMT Usage/Full Body

Rates of osteopathic/chiropractic manipulation more than doubled from 2010 to 2015 [27]. The most common diagnosis treated by these modalities was “unspecified disorders of the back” [27]. In a study of service members with chronic pain, Meerwijk et al. compared those who received NPT (which included OMT) with those who received pharmacologic treatment. The group receiving NPT, which included OMT, had lower rates of drug use disorder, suicidal ideation, and self-inflicted poisonings [28]. Additionally, the NPT group had a 0.1% lower death rate [28].

Limitations

Due to the nature of a scoping review, this study is limited by our inclusion and exclusion criteria and the available literature. After reviewing the articles, only nine met the inclusion criteria. Of these, four studies had small sample sizes, and two obtained their data from the same retrospective chart review [20,21,23-26]. 

Only two of the nine studies were randomized controlled trials (RCTs), while the remaining seven were either case reports, case series, or cohort studies. These non-RCT studies provide weak evidence for a causal relationship between the OMT patients received and symptom improvement.

Another limitation seen in four out of the nine articles was the use of nonspecific terms like NPT or CAM in the treatment protocols [25-28]. These umbrella terms include osteopathic manipulation, chiropractic manipulation, acupuncture, massage, music therapies, and art therapies. In these studies, it is unclear to what extent osteopathic manipulation was performed or osteopathic techniques were used. The inclusion of other NPTs introduces confounding variables that complicate the study results. 

Confounding variables also arise from differences in the frequency and timing of OMT treatments among the studies. While most studies did not specify treatment frequency or timing, three of the nine articles indicated that patients received OMT once per week [20,22,23]. Of these, Cruser et al. specifically studied active-duty personnel. These individuals have varying activity levels throughout the week based on their specific jobs, which could influence pain reporting between osteopathic treatments and impact the results of study questionnaires.

Finally, OMT research is inherently limited by issues with blinding and control. Due to the hands-on nature of the treatment and the need for accurate diagnosis, provider blinding is challenging. To increase validity, some studies use a sham group, where the provider performs a manipulation that mimics OMT. However, this technique may be noticeable to patients, especially those with prior experience with OMT, thus reducing the effectiveness of blinding. One study used a sham treatment control group [21], while another opted not to use a sham group, limiting the ability to determine whether OMT itself influenced the study results or if the results were simply due to the act of provider contact [22]. 

Implications for research

Overall, this review suggests that OMT can positively impact patient pain perception and symptom management across various pathologies in the military. Multiple studies included in this review were either case reports, pilot studies, or had small sample sizes. Future research should focus on randomized controlled trials with larger patient populations to strengthen the evidence regarding the effectiveness of OMT in the military community. On top of that, NPTs should be studied individually to minimize confounding variables and increase clarity in the literature. Employing these approaches may increase the utilization of OMT, reduce reliance on pharmacologic pain management, and improve patient outcomes. 

Given the large military population and its presence in the United States, it is clear that the US Military needs more than the mere nine studies currently available on the efficacy of OMT. There are numerous benefits to utilizing OMT, including, but not limited to, lower healthcare costs, higher physical demands on service members, patient preference for nonpharmaceutical treatment options, and the fact that OMT is a valid treatment option. 

## Conclusions

This paper assessed the current use of OMT in military medicine and, more importantly, highlighted the need for larger-scale studies to confirm its effectiveness in the military. Initial studies show promising results, including reduced pain levels and lower healthcare costs. However, OMT must be studied as a single variable to better isolate its effects. Large-scale studies would greatly benefit the military population by evaluating the potential of OMT as a common medical therapy in military medicine.
